# Electrophysiological evidence for sensitization effects elicited by concurrent social threats

**DOI:** 10.1038/s41598-023-39456-0

**Published:** 2023-07-28

**Authors:** Michael Niedeggen, Xu Fang, Yu-Fang Yang, Rudolf Kerschreiter

**Affiliations:** 1 Division of Experimental Psychology and Neuropsychology, Department of Education and Psychology, Freie Universität Berlin, Habelschwerdter Allee 45, 14195 Berlin, Germany; 2 Division of Social, Organizational, and Economic Psychology, Department of Education and Psychology, Freie Universität Berlin, Berlin, Germany

**Keywords:** Psychology, Neuroscience, Cognitive neuroscience, Social behaviour, Social neuroscience

## Abstract

Experiencing a social threat, such as social exclusion, is a painful event. In contrast to previous studies providing insight into the processing of a single short-termed threat, we exposed healthy individuals to the simultaneous onset of different social threats. This approach allowed us to track whether these threats are processed independently—or whether they interact in a common system. Using a virtual ball-throwing game (Cyberball), electrophysiological (event-related brain potentials, ERPs) and behavioral (self-reports) responses were collected. We assigned undergraduates to three experimental groups: single threat exclusion (n = 24), single threat loss of control (n = 26), and joint onset of both threats (dual-threat, n = 25). Self-reports indicated an increase in threats (i.e., in perceived exclusion and loss-of-control) in the latter group. The ERPs disentangled the neural responses to each threat: In the dual-threat group, the amplitudes of the P3 responses to exclusionary and intervention events were enhanced. This indicates that individuals are sensitized to each of the threats when the other threat is present simultaneously. Our findings support the theoretical notion of a common cognitive system responding to violations in subjective expectations.

The assurance of fundamental social needs defines a prerequisite of mental health and well-being^1^. Besides safety and acknowledgment, human beings long for social belonging and control^2,3^. Threats to these social needs are commonly reported as stressful and anxiety-provoking, and trigger palliative responses^4^. Previous studies demonstrated that behavioral and physiological responses to social threats can be modulated, for instance by social primes^5–7^. The present research aims to answer a more fundamental question, namely whether the processing of a social threat is modulated if it is accompanied by the experience of another—independent—social threat.

A direct test of this idea requires a setup that allows for the independent experimental manipulation of different social threats in a single experimental setting. We took advantage of the established Cyberball paradigm^8^: Here, the participant is informed to be connected to two (putative) co-players represented as avatars on a computer screen. The participant, also represented as an avatar, is instructed to visualize the ball-throwing game mentally and to pass the ball—when received—to one of the co-players.

The Cyberball paradigm is commonly used to induce a threat to the need for belonging: If the probability of ball reception is reduced, participants reliably report feeling of exclusion^9^. A slight modification of the paradigm induces a selective threat to the need for personal control—here defined as the ability to select options^10^: In this recently introduced *intervention* Cyberball^11^, the participant remains included in the game, but the participant’s decision on the recipient of the own ball throw is casually overruled by a putative supervisor. The specificity of the experimental manipulation has been validated by commonly used self-report scales (NTQ, Need-Threat-Questionnaire,^12^). Here, the unpredictable intervention did not affect the feeling of belonging—but elicited a selective threat to control.

Notably, although the need to belonging and the need for control are both basic human needs, they also differ in several respects: For example, belonging is closely tied to social interaction and in the experimental situation threatened by the behavior of the co-players. In contrast, a loss-of-control is not necessarily associated with social interaction but would also be experienced as a threat in a non-social context. Interestingly, despite this difference, a common affective and behavioral response pattern has been reported: Threats to social needs trigger aversive arousal and compensatory efforts which reflect themselves in assimilation^13^ or affirmation^14^. This similarity is in line with overarching uncertainty models, such as the meaning maintenance model^15^ which predicts that violations of subjective beliefs and expectations motivate people to dispel the resulting aversive arousal.

Uncertainty models imply that the processing of different threats relies on the activation of a common neural network^16^. Although the findings of neuroimaging studies are controversially discussed^17^, electrophysiological studies indicated that different threats trigger comparable cognitive processes: Both, the onset of exclusion^18,19^ and the onset of a loss-of-control^11^ elicit an increase in amplitude of a well-known component in the event-related brain potentials (ERPs), the P3. The increase in amplitude—further labelled as P3-effect—does not merely reflect a change in the probability of relevant events^20^, but critically relies on a violation of the participants’ subjective expectancies on social participation and control, respectively^21,22^. In line with this idea, experimental manipulation of the level of subjective expectation, for instance by a differential self-assignment of social power^23^, also affects the expression of the P3-effect. Importantly, this moderation can be consistently observed for a violation of expected participation (exclusion Cyberball,^6^ or expected control (intervention Cyberball,^11^). In other words, changes in the P3 reflect deviances in a subjective belief system that seems to be related to a common cognitive system^24^.

However, it is unclear whether the experience of a social threat affects the sensitivity to a different social threat. Experimental approaches mostly follow a ‘social priming’ approach: The effect of a precedent condition on the processing of a following threat is examined^5,7,25^. In contrast, combining the established exclusionary Cyberball setting with the intervention option^11^ allows us to examine the interaction of two threats in one setting. Since both threats are related to separate events in the ball-throwing game, the reduced probability of ball reception (exclusion) and causal interventions in the participants’ decision (control), ERPs can be applied to disentangle the immediate neural responses. Specifically, the P3 will provide a probe for expected participation and decisional autonomy, respectively.

Based on this experimental setup, we asked: Is the sensitivity to social exclusion increased if a loss of control (here: intervention) is experienced simultaneously, and vice versa? We hypothesized that a selective threat to the need for “belonging” (induced by exclusion) and a selective threat to the need for “control” (induced by intervention) will predominantly affect the corresponding scales in the post-hoc questionnaire (NTQ). A joint onset of both threats was assumed to increase the effects on both scales. This hypothesized interaction effect is proposed to rely on the sensitization of a common cognitive system involved in the processing of different inconsistencies^16^. The corresponding neural dynamics activated by the joint onset of two threats will affect the expression of the P3-effect reflecting violations of subjective expectancies^26^ . Correspondingly, we hypothesized that the P3-effect related to the transition-to-exclusion is more expressed if a loss of control is experienced simultaneously, and vice versa.

The implication of the hypothesized interaction is of practical relevance: Negative life events which contribute to the development of anxiety and depression disorder^27^ are usually defined by the threat to multiple needs.

## Methods

The experimental procedure was approved by the Ethics Committee of the Department of Education and Psychology, FU Berlin (No.006.2019, May 15, 2019) and written informed consent was taken/obtained in all participants according to the Declaration of Helsinki.

All data (self-reports, pre-processed EEG data), program, and analysis codes are available in a data repository (https://osf.io/kad5t/?view_only=28a0f79c140f443aabaa2889bc3e296d).

In this manuscript, we report all measures and effects of the experimental manipulations. The exclusion criteria are detailed below.

### Participants

The sample size was determined a priori using G*Power^28^. The calculation was focused on the effect hypothesized for the ERP data, namely the P3-effect elicited by a transition to a social threat. It was assumed that a transition to exclusion and intervention, respectively, will induce a P3-effect. A joint onset of threats will lead to a significant increase of the P3-effect. Consequently, our analysis was focused on an interaction effect of a between-factor “threat combination” (three experimental groups) and a within-factor “experimental block” (two blocks). Based on a previous study with a similar design^6^, we assumed a corresponding medium-sized effect (*f* = 0.20 adjusted to the taxonomy of Cohen) for the hypothesized interaction. To detect such an effect with a power of 80% and an alpha at 0.05, a sample size of 66 participants was required.

As indicated in previous ERP cyberball^29,30^, a rejection rate of approximately 20% had to be considered. Accordingly, a total sample of 93 participants (71 female, 22 male, age range: 18—36 years) was recruited. All participants were undergraduate students from the FU Berlin (see Sect. “Limitations”). Participants were randomly assigned to the three experimental groups (EG_excl_: exclusion-only, EG_int_ intervention-only, EG_combined_: exclusion + intervention). Following a rigorous artifact rejection in EEG analysis (criteria are described in section: Data analysis), data of 18 participants (16 female, 2 male, mean age: 23.9, SD: 5.29) had to be rejected. The final sample comprised 75 participants (55 female, 20 male, mean age: 23.13, SD: 4.65). Experimental group 1 (EG_excl_) included 24 participants (16 female, 8 male, mean age: 24.71, SD: 5.60), experimental group 2 (EG_int_) included 26 participants (18 female, 8 male, mean age: 22.42, SD:4.25), and experimental group 3 (EG_combined_) included 25 participants (21 female, 4 male, mean age: 22.36, SD:3.77).

### Task and design

All participants were provided with a cover story: According to the written instructions, they took part in a study on visual imagination abilities. While playing the upcoming ball throwing game, they had to imagine either a beach scenario (“Imagine that you and your fellow players play the ballgame on the beach.”) or a meadow scenario (“Imagine that you’re playing the ballgame on a meadow.”). To support this cover story, a corresponding questionnaire had to be filled out (Vividness of Visual Imagery Questionnaire,^31^).

**Figure 1:**
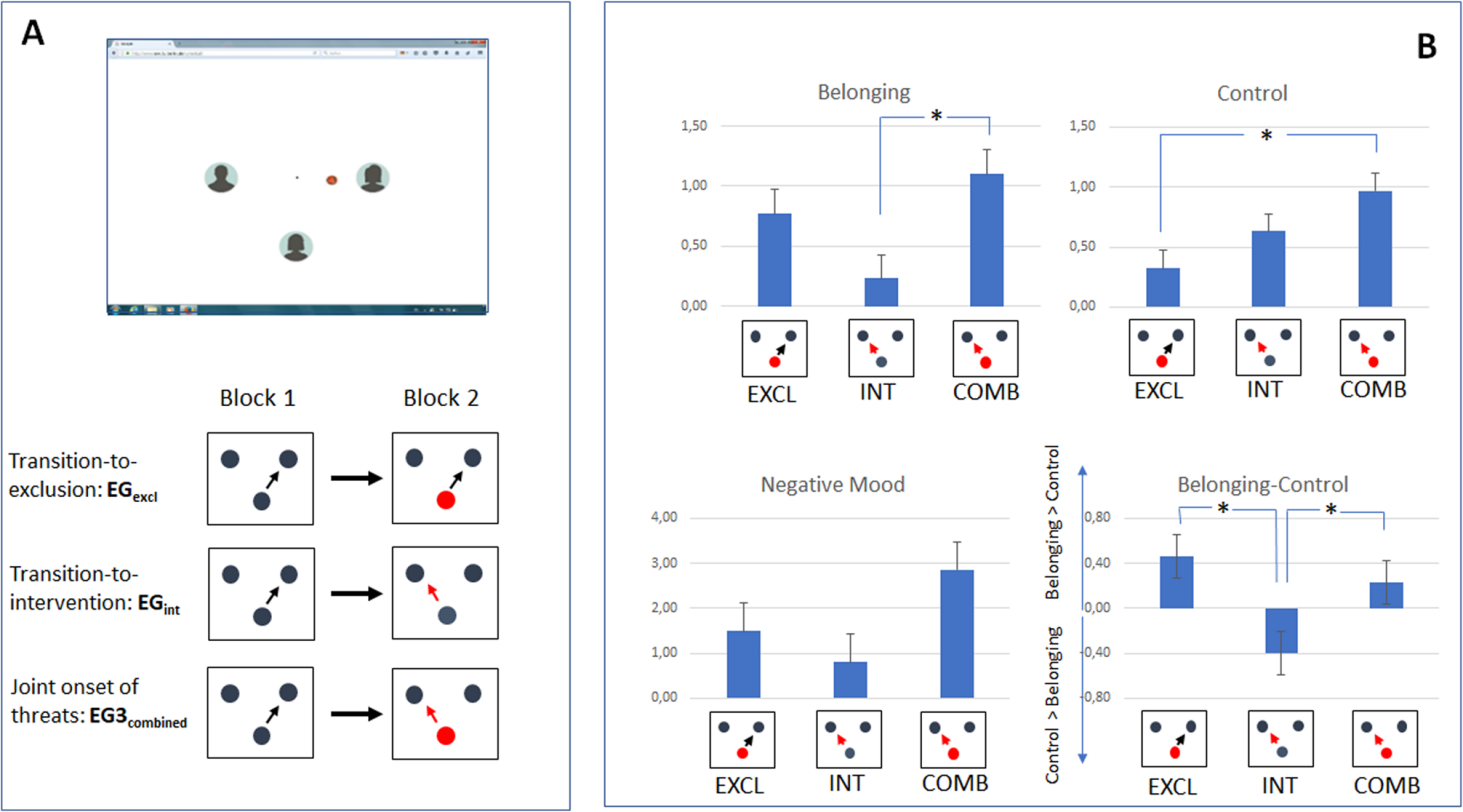
(**A**) Setup and Design. On the computer screen, three avatars represented the participant (middle position) and the two putative co-players. The symbol of the ball in spatial proximity to one avatar signaled the possession of the ball. Each participant ran through two blocks of the Cyberball game. In block 1, all participants were included and no interventions occurred. In block 2, participants were either partially excluded (EG_excl_, indicated by red dot), overruled (EG_int_, indicated by red arrow), or received both threats (EG_combined_, indicated by red dot and arrow). (**B**) Effects on the self-report scales. Mean values refer to the difference between block 2 and block 1. In the NTQ scales of interest (belonging, control) as well as in negative mood, the onset of joint threats (EG_combined:_ red dot and arrow) induced more-pronounced effects. The difference values computed for ratings on the scales ‘belonging’ and ‘control’ confirmed the specificity of the experimental manipulation. The asterisk indicates a significant difference between experimental groups.

The experimental procedure was programmed in PsychoPy2 (version V1.85.6^32^) and was based on a cyberball game adapted for ERP recording^18^. The setup of the following cyberball game included the intervention condition^11^. Figure 1A depicts the visual display: The participant was putatively connected with two co-players via the internet, and all players were represented by three avatars on the computer screen (7° × 7° at a viewing distance of 120 cm). The avatar of the participant was always placed centered on the computer screen horizontally and below the avatars of the putative co-players vertically. The vertical position of the avatars of the two putative co-players was centered. The spatial distance between the avatars was held constant (3°) in each of the experimental conditions. To strengthen the intended binding, participants were asked to select an avatar of their choice before the game started^33^.

The ball was represented by a corresponding symbol on the display. If it was presented in spatial proximity to the participants' avatar, it signaled ball possession. In this case, it had to be forwarded to one of the co-players by pressing a corresponding button on a keyboard (‘G’: ball to left co-player, ‘H’: ball throw to right co-player). Following the button pressed, the ball vanished for 500 ms and re-appeared in spatial proximity to a co-player’s avatar. To simulate the temporal variability of a decisional process of the co-players, their ball possession lasted randomly between 400 and 1.400 ms. Then, the ball vanished for 500 ms and reappeared near the position of another avatar. Participants were allowed to follow the position of the ball (range of ocular movements: max. 2°) and to make eye blinks, if necessary. Otherwise, eye movements should be avoided. The participant’s head was stabilized by a chin rest.

The practice trials (10 ball throws, ball reception probability: 0.3, no intervention) were followed by two experimental blocks each comprising 250 trials. Completion of one block took approximately 10–12 min. The variance is due to the fact that speeded response was not required. Between the successive blocks, a short break (3 min) was included. To support the cover story, each experimental block was preceded by a picture of the environment to be visualized (meadow or beach, presented in counterbalanced order) and the corresponding written instruction. Furthermore, all participants—independently of the assignment to an experimental group—were informed that an independent ‘supervisor’ might intervene in the decision of the players. Interventions will result in ball possession of the *non-intended* co-player: In other words, the ball will not be forwarded to the co-players addressed by the corresponding button press. It was also mentioned that the supervisor is not one of the co-players involved in the game. As mentioned above, this manipulation was intended to threaten the need for control selectively^11^.

In block 1 of the cyberball game, social involvement and control were comparable for all experimental groups: Ball reception of the participant was set at 33% defining an inclusionary condition (defining 82 trials for EEG analysis). The supervisor did not intervene and intervention probability was set at 0% defining high personal control (82 trials). In block 2, participants assigned to EG_excl_ were partially excluded, and the probability of ball reception was slightly reduced to 22% (55 trials), but the probability of intervention remained 0% (55 trials). Participants in EG_int_ were still included (82 trials), but the supervisor casually overruled the participants’ decision on the recipient of the ball throw with an intervention probability of 30% (57 trials). In EG_combined_, both threats were combined: ball reception probability was reduced to 22% (55 trials) and intervention probability was set at 30% (38 trials). Please note that the number of trials provided in brackets refers to the number of events foreseen for EEG analysis: The event “ball reception of the participant” served as a probe for the processing of exclusion, and “ball reception of the intended co-player” served as a probe for the processing of intervention.

Following the completion of the second block of the cyberball game, participants had to estimate the frequency of ball receptions and intervention in both blocks (manipulation check). Moreover, they had to fill out the NTQ^9,34^ measuring the threat to four social needs (belonging, self-esteem, meaningful existence, and control). Negative mood was measured using an eight-item adjective list (e.g. “I felt sad” or “I felt angry”,^35^). Additional four items were related to two forms of power, namely social power (two items, i.e. *I felt in charge of others*) and personal power (two items, i.e. *I felt independent*,^36^). In all self-report scales, participants had to estimate the change in threat, mood, or estimated power, respectively, on a 7-point Likert scale (“-3” = much stronger in block 1 to “3” = much stronger in block 2). The relative rating reduces the mnestic affordance, and has been successfully applied previously^25^. Note that the results section will focus on the social needs selectively addressed in our experimental design (belonging, control), and on negative mood. The data of the remaining self-reports—which did not reveal a significant interaction of the experimental factor ‘group’—can be found in the repository.

Following the completion of the questionnaire, participants were debriefed: They were informed that the experiment focused on the effects of social exclusion and intervention. The behavior of the virtual co-players and supervisor followed an experimental protocol. The necessary deception regarding the goals of the experiment required that written consent has been asked for at the beginning and the end of the experiment. The completion of the experiment—including the electrode montage—required approximately 90 min.

The choice of a within-participant design is largely based on the electrophysiological methods included in this study: To estimate the ERP effects of exclusion, respectively a loss-of-control, as a reliable baseline must be provided (here: block 1). This baseline allows us to estimate the effects of a transition to a social threat. The use of a between-participant design will result in a marked reduction in sensitivity^26^.

### EEG recording

EEG was recorded at eight active electrode sites (AFz, Fz, F3, F4, Cz, Pz, P7, P8) using Ag/AgCl electrodes embedded in an elastic cap (EASYCAP, Herrsching, Germany; Brainmps amplifier, BrainProducts, Gilching, Germany). Impedance of active electrodes was kept below 10 kΩ and the signals were referenced to linked earlobes, with FCz serving as ground. EEG data were recorded continuously and sampled at 500 Hz. Online, the EEG signal was band-pass filtered (0.1–100 Hz) and the effect of AC hum was reduced by applying a notch filter (50 Hz). Ocular artifacts were controlled by recording vertical and horizontal electrooculograms (EOG).

‘Vision Analyzer’ (Version: 2.1, Brain Products, Gilching, Germany) was applied for offline EEG analysis. Following a bandpass filtering (0.3–30 Hz, 24 dB/Oct) of the EEG data, EEG signals were epoched (−100 to 800 ms) separately for the two experimental blocks based on the two events defined above: ERPs to the event “ball reception of the participant” respond to exclusion and ERPs to the event “ball reception of the intended co-player” respond to intervention. The P3 response to the event ‘ball reception of the participant’ served as a reliable probe of the participants' state of expectancy^22^. Its dynamics can be related to systematic changes in state expectancy^26^. This process is the subject of our hypothesis. The ERP responses triggered by the event “ball reception of the intended co-player” are less established^11^, but follow the same rationale: The transition to a loss ofcontrol (Block 1 to Block 2) reduces the probability of events, and triggers a violation of expectancy.

EOG channels of the baseline-corrected trials (−100 to 0 ms) were automatically controlled for ocular artifacts (EOG > 50 μV). Trials with ocular artifacts were rejected from the analysis. In a second semi-automatic artifact rejection, trials were first marked if an amplitude threshold was exceeded (EEG > 75 µς), and rejected if this was due to high alpha activity or movement artifacts. In a final manual correction, EEG trials were analyzed for slow linear drifts affecting the baseline period, or high-frequency bursts. Following the rigorous artifact rejection, trials were averaged separately for the different experimental blocks. As in previous studies, data of a participant were discarded from analysis if an averaged ERP signal relied on less than 15 trials. For the event ‘ball reception of the participant’, the mean number of trials included in the analysis was 46.7 (SD: 12.5) in block 1 (rejection rate: 43%), and 29.7 (SD: 13.72) in block 2 (rejection rate: 46%). For the event ‘ball reception of the intended co-player’, the mean number of trials included in the analysis was 51.8 (SD: 12.8) in block 1 (rejection rate: 37%), and 26.1 (SD: 9.13) in block 2 (rejection rate: 48%).

In a subsequent analysis, the number of EEG segments in the condition “ball reception” in block 1 was adjusted to the number of segments in the condition “ball reception” in block 2 by random selection. This procedure was also applied to the condition ‘ball reception of the intended co-player’. The critical interactions (“Group” x “Block”) reported in the following do also hold if one considers the differences in the signal-to-noise ratio between the ERPs in block 1 and block 2. The corresponding data are available in the data repository, and the results of analysis in the supplement.

### Data analysis

#### Questionnaire data

In a first manipulation check, we tested whether the changes in the probability of ball reception and intervention were remarked by the participants. To this end, the estimated frequency of ball reception was analyzed by running a two-factorial ANOVA including the between-factor ‘group’ (EG_excl_ vs EG_combined_: both including a transition-to-exclusion) and the within-factor ‘block’ (block 1 vs block 2). Accordingly, the estimated frequency of intervention was analyzed including the between-factor ‘group’ (EG_int_ vs EG_combined_: both including a transition-to-intervention) and the within-factor ‘block’ (block 1 vs block 2).

To test whether the experimental manipulations (exclusion and intervention, respectively) elicited a specific pattern of social threat, the difference score between the ratings of the NTQ scales ‘belonging’ and ‘control’ was computed for each participant (Belonging—Control). The transition to exclusion was assumed to elicit a positive value, whereas the increase in the intervention frequency should trigger a negative value of the difference score. The differences scores were analyzed by running a one-factorial ANOVA including the between-factor ‘group’ (EG_excl_ vs EG_int_ vs EG_combined_). Significant differences between the single-threat groups (EG_excl_ vs EG_int_) will confirm that the exclusionary cyberball primarily affects the need for ‘belonging’ whereas the intervention cyberball primarily affects the need for ‘control’.

The three single scales of interest (belonging, control, and negative mood) were analyzed separately by running a one-factorial ANOVAs including the between-factor ‘group’ (EG_excl_ vs EG_int_ vs EG_combined_). Please note, that the self-reports are already based on the differential rating (change from block 1 to block 2, see above).

In the final step of the analysis, the correlation of the NTQ self-report scales ‘belonging’ and ‘exclusion’ was tested (Spearman correlation coefficient) separately for the experimental groups. Additionally, the modulation effect of negative mood was considered by computing the partial correlation coefficient. For statistical analysis, SPSS (version 27, IBM) was used. In case of a significant interaction in an ANOVA, post-hoc comparisons were run.

#### ERP data

To determine the time regions for statistical analysis, grand-averaged ERPs responses to the event ‘ball reception/participants’ and ‘ball reception/intended co-player’ were computed for the difference waves between block 2 and block 1—independently of group assignment. The difference waves were chosen to extract the effect of the transition. For the resulting ERPs, the global field power index was determined, and local maxima in the time range 300 to 500 ms were identified. Please note that the choice of the time range relies on previous results: Previous research on the exclusionary cyberball^37^ reported latencies of P3 amplitudes as triggered by ball reception between 320 and 420 ms. The same applies to the P3 responses in the intervention cyberball^11^. As for the condition ’ball reception/participant’ (serving as a probe of expected participation), a first local maximum was identified at 320 ms, followed by a second—more sustained—maximum starting at 390 ms. Considering interindividual differences, the first temporal window was defined in the time range 290 to 350 ms referring to an early P3 response, and the second temporal window in the time range 360 to 420 ms window referring to a late P3b-like response. As for the condition ‘ball reception/intended’ (serving as a probe for the expected control), a first local maximum was identified at 220 ms. A subsequent local maximum was identified at 370 ms, and was covered by the corresponding temporal window extending from 340 to 400 ms.

For each of these time windows defined above, mean amplitudes were computed for the participants’ ERP data separately for the experimental condition ‘block’ and electrodes. Due to the more sustained expression of the P3 component, a reliable peak identification of the components was not possible.

Besides of the a priori defined analysis of the P3, the grand-averaged data also indicated that two more components—previously related to the processing of social exclusion—were expressed in our data: For both events, ‘ball reception of the participant’ as well as ‘ball reception of the intended co-player’, a transient negativity at about 170 ms was identified. This N2 response has been previously related to conflict monitoring (for review, see^37^). Based on the global field power, a temporal window from 140 to 200 ms was defined for this component. For the event, ‘ball reception of the intended co-player’, the grand-averaged ERPs indicated a transient positivity at about 220ms. According to the latency, the positivity qualifies as a P2 component which has been related to changes in attentional allocation^38^, as well as to reward processing^39^. Based on the global field power, a temporal window from 190 to 250 ms was defined for this component.

The exported amplitude data of the ERP components were analyzed separately running 3 × 2 × 4 ANOVAs, including the between-participant factor ‘group’, and the within-participant factors ‘block’ and ‘electrode’. The latter factor comprised the four midline electrodes (AFz, Fz, Cz, and Pz) to account for the shift of topography in the caudal dimension. The ANOVA results are reported with Greenhouse–Geisser corrected degrees of freedom and p-values. Post-hoc comparisons were motivated by significant interactions of the experimental factors.

## Results

### Manipulation check and validation of threat-specific response in self-reports

The two experimental groups experiencing a transition-to-exclusion noticed a reduction in ball reception probability: In EG_excl_ (exclusion-only), estimated probability for ball reception dropped from 34.2% (SD: 12.4) in block 1 to 18.6% (SD: 5.2) in block 2. In EG_combined_ (exclusion + intervention), a corresponding effect was observed between block 1 (29.5%, SD: 6.5) and block 2 (16.7%, SD: 6.8). The effect of the factor ‘block’ was significant, F(1,47) = 96.96, p < 0.001, η_p_^2^ = 0.674—but was not modulated by the factor ‘group, F(1,47) = 0.81, *p* = 0.374, η_p_^2^ = 0.017. In the experimental group not experiencing a transition-to-exclusion (EG_int_), estimated ball reception remained stable (Block 1: M = 32.1%, SD = 9.6, block 2: M = 28.8%, SD = 7.9).

For the two groups experiencing a transition-to-intervention corresponding effects were observed: EG_int_ (intervention-only) remarked an increase in intervention probability from block 1 (5.5%, SD: 7.7) to block 2 (26.6, SD: 18.2). In EG_combined_ the perceived increase from block 1 (4.2, SD: 8.1) to block 2 (33.6, SD:16.2) was slightly more expressed. As expected, the effect of the factor ‘block’ was significant, F(1,49) = 131.22, *p* < 0.001, η_p_^2^ = 0.728. However, the effect was not significantly modulated by the factor ‘group’, F(1,49) = 3.18, *p* = 0.081, η_p_^2^ = 0.061. In the experimental group not experiencing a loss-of-control in the second block (EG_excl_), estimated interventions were slightly enhanced (block 1: M = 11.3%, SD = 18.6, block 2: M = 18.9%, SD = 7.9).

To validate whether the experimental manipulation in our three experimental groups leads to a specific threat of belonging and control, we first analyzed the specificity of the response in the NTQ questionnaire. To this end, the difference score between the rating for the two scales, belonging and control, was computed in each participant (see Table 1, Fig. 1B). The descriptive values indicated a differential response pattern depending on the manipulation: Specifically, a threat to the need for ‘belonging’ was more expressed in EG_excl_, whereas a threat to the need for ‘control’ was more pronounced in EG_int_. A joint onset of the threats (EG_combined_) induced a slight dominance of a threat to the need for ‘belonging’. The ANOVA signaled a significant difference between the groups, F(2,72) = 5.56, *p* = 0.006, η_p_^2^ = 0.134. Post-hoc comparisons confirmed a differential response in the two scales when comparing EG_excl_ and EG_int_, F(1,48) = 8.86, *p* = 0.005, η_p_^2^ = 0.156., and EG_int_ and EG_combined_, F(1,49) = 6.06, *p* = 0.017, η_p_^2^ = 0.110. No differences were obtained when comparing EG_excl_ vs EG_combined_: F(1,47) = 0.801, *p* = 0.375, η_p_^2^ = 0.017. The results confirm that the exclusion condition primarily affects the response on the scale ‘belonging’, and that the intervention condition primarily affects the scale ‘control’.

**Table 1:** Descriptive statistics for the self-reports in the NTQ and negative mood scale separated for the three experimental groups: Data for the scales ‘threat to belonging’, ‘threat to control’, and ‘negative mood’ refer to the differential rating (block 2 vs block1) with positive values indicating a higher expression in block 2. Significant effects depending on group assignment were obtained for the scales ‘belonging’ and ‘control’. The difference value (rating for scales ‘belonging’—‘control’) indicates the specificity of the experimental manipulation which is confirmed by a significant difference between the groups EG_excl_ and EG_int_. The first line in each cell provided the mean and standard error of mean (SEM), and the second line provided the upper and lower limits of the confidence intervals (95%). The last two rows show the correlation between the two NTQ scales. To account for a mediating effect of negative mood, the last row shows the partial correlation (r_BEL, CONT-NM)_. (*EG_excl_ = Experimental Group / Exclusion, **EG_int_ = Experimental Group / Intervention, ***EG_combined_ = Experimental Group / Combined Exclusion and Intervention).

	Means, SEM, Confidence interval (95%)				Correlation	
	Belonging	Control	Neg Mood	Δ(Belong-Control)	r_BEL,CONT_	r_BEL,CONT-NM_
EG_excl_*	0.77 (0.20) 0.37, 1.18	0.32 (0.15) 0.03, 0.61	1.50 (0.63) 0.24, 2.76	0.46, 0.19 0.07, 0.84	0.456 *p* = 0.025*	0.040 *p* = 0.855
EG_int_**	0.23 (0.20) − 0.16, 0.62	0.63 (0.14) 0.35, 0.91	0.81 (0.61) − 0.40, 2.02	− 0.40, 0.19 − 0.03, − 0.77	0.239 *p* = 0.239	0.046 *p* = 0.827
EG_combined_***	1.10 (0.20) 0.70, 1.50	0.97 (0.15) 0.66, 1.26	2.86 (0.62) 1.63, 4.10	0.23, 0.19 − 0.15,0 .60	0.558 *p* = 0.004*	0.502 *p* = 0.012*

### Self-reports

As depicted in Fig. 1B and Table 1, the expression of the self-reported threat to the different needs was differently affected by the experimental manipulation.

As for the scale ‘belonging’, the mean threat was more expressed by a transition to exclusion, and this effect was further increased by the joint onset of both threats in block 2 (EG_combined_ > EG_excl_ > EG_int_). Significant differences between the groups were signaled by the ANOVA, F(2,72) = 4.90, *p* = 0.010, η_p_^2^ = 0.120. Post-hoc comparisons revealed a significant difference exclusively between EG_int_ and EG_combined_, F(1,49) = 10.19, *p* = 0.002, η_p_^2^ = 0.172. In contrast, no significant difference was observed for the remaining contrasts: EG_excl_ and EG_int_, F(1,48) = 3.55, *p* = 0.066, η_p_^2^ = 0.069, and EG_excl_ and EG_combined_, F(1,47) = 1.22, *p* = 0.275, η_p_^2^ = 0.025.

A corresponding pattern was observed for the scale ‘control’: The mean threat was increased by the onset of intervention, but the effect was amplified by the joint onset of two threats (EG_combined_ > EG_int_ > EG_excl_). The significant effect in the ANOVA, F(2,72) = 5.01, *p* = 0.009, η_p_^2^ = 0.122, triggered post-hoc comparisons. Here, a significant difference was confirmed between EG_excl_ and EG_combined_, F(1,47) = 8.12, *p* = 0.006, η_p_^2^ = 0.147. Other pairwise comparisons were not significant: EG_excl_ and EG_int_, F(1,48) = 2.30, *p* = 0.136, η_p_^2^ = 0.046, and EG_int_ and EG_combined_, F(1,49) = 3.67, *p* = 0.061, η_p_^2^ = 0.070.

Since a threat to social needs is associated with negative arousal, the effects of the experimental manipulation on the negative mood were also considered. The values indicated that exclusion elicited a stronger negative mood as compared to a loss of control and that this effect was enhanced by the experience of both social threats (EG_combined_ > EG_excl_ > EG_int_). The ANOVA, however, did not indicate significant differences between the three experimental groups, F(2,72) = 2.88, *p* = 0.063, η_p_^2^ = 0.074.

In the final step of the analysis, the correlations between the expression of the reported threats to social needs were examined (r_BEL,CONT_). Moreover, it was examined whether the relationship between belonging and control was moderated by negative mood (partial correlation coefficient: r_BEL,CONT.NM_). In both experimental groups experiencing the transition to a specific threat, the correlation between the self-reported threats to ‘belonging’ and ‘control’ was markedly reduced if negative mood was considered (EG_excl_: r_BEL,CONT_ = 0.456 vs r_BEL,CONT-NM_ = 0.040, EG_int_: r_BEL,CONT_ = 0.239 vs r_BEL,CONT-NM_ = 0.046). In contrast, the joint onset of both threats in EG_combined_ elicited a significant correlation between the self-reported threats to ‘belonging’ and ‘control’ (r_BEL,CONT_ = 0.558, *p* = 0.004) that was only slightly reduced if the factor ‘negative mood’ (r_BEL,CONT-NM_ = 0.502, *p* = 0.012) was considered.

### ERP effects: Ball reception of the participant

**Figure 2:**
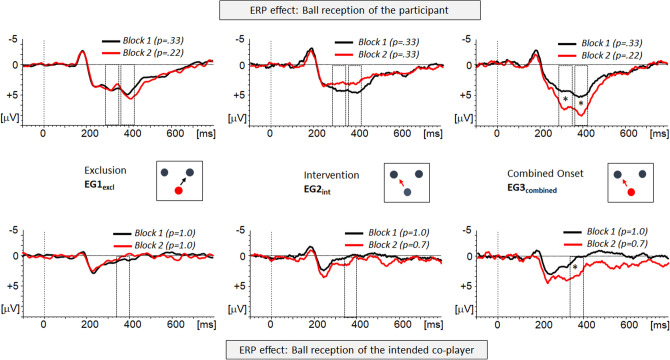
ERP effects at centro-parietal electrode leads (electrodes clustered). Upper vs. lower panel: The upper panel contrast the ERPs elicited by the event “ball reception of the participant” in block 1 (black trace) and block 2 (red trace). The probability of ball reception is provided in the figure. The lower panel presents the ERPs elicited by the event ‘ball reception of the intended player’ following the participants’ ball possession. Block 1 (black trace) and block 2 (red trace) are contrasted, and the probability of the intended event is provided in the figure (*p* = 0.7 refers to an intervention probability of 0.3). Left vs. central vs. right panel: The ERP effects for both events are depicted separately for the three experimental groups (EG_excl_: left panel, EG_int_ central panel, EG_combined_ right panel). The time windows considered for the analysis of the P3 effects are highlighted. The asterisks indicate that the P3 effect (block 2—block 1) is significantly enlarged in the group EG_combined_ as compared to both, EG_excl_ and EG _int_.

To register the processes elicited by a transition-to-exclusion, and to probe the state of the expected participation, the change in the P3 responses elicited by the event ‘ball reception of the participant’ has been used^22^. Figure 2 (upper panel) shows the effects for the three experimental groups: Following a transient negativity at 180 ms, a sustained positivity extending from 200 to 500 ms was observed consistently. The global field power indicated two maxima in this time range which were analyzed (see Sect. “Methods”): An early P3 range (290–350 ms) followed by a late P3 segment (360–420 ms) covering the P3 maximum. Moreover, the first negative (N2) was analyzed a posteriori.

The amplitude level of early P3 was found to be enhanced when comparing block 1 and block 2 in EG_combined_ (see Table 2). In the other groups defined by the onset of a single threat, the level of the P3 amplitude remained unchanged (EG_excl_), or was slightly reduced (EG_int_). The ANOVA did not reveal a significant main effect of the factor ‘block’, F(1,72) = 3.61, *p* = 0.061, η_p_^2^ = 0.048, but confirmed a significant interaction of the factors ‘group’ and ‘block’, F(2,72) = 18.68, *p* < 0.001, η_p_^2^ = 0.342. In the following post-hoc-tests, the expression of the P3-effect (block 2 vs. block 1) was compared between the blocks. Confirming the descriptives, the P3-effect was significantly larger in EG_combined_ as compared to EG_excl_, F(1,47) = 13.44, *p* < 0.001, η_p_^2^ = 0.222, and also as compared to EG_int_, F(1,49) = 55.01, *p* < 0.001, η_p_^2^ = 0.529. No differences were found between EG_excl_ and EG_int_, F(1,48) = 2.74, *p* = 0.104, η_p_^2^ = 0.054. Please note that the critical interaction was not modulated by the position of the midline electrodes, ‘block’ x ‘group’ x ‘electrode’ F(2.74,98.65) = 2.28, *p* = 0.089, η_p_^2^ = 0.062.

**Table 2:** Mean amplitudes (µV), SEM (in brackets) and confidence intervals (lower and upper limits, 95%) of the ERP effects. Data are separated for the ERP responses to the events “ball reception of participant’ and “ball reception of intended co-player”. For each of these events, the values are provided for the time ranges related to the relative maxima.

	Event: ball reception participant		Event: ball reception intended co-player	
	290–350 ms Early P3	360–420 ms Late P3	190–250 ms P2	340–400 ms P3
EG_excl_ Exclusion-only				
Block 1	3.80 (0.47) 2.86, 4.74	4.22 (0.65) 2.93, 5.51	2.06 (0.35) 1.37, 2.76	0.70 (0.33) 0.40, 1.36
Block 2	3.77 (0.62) 2.55, 5.00	4.77 (0.75) 3.28, 6.26	1.95 (0.43) 1.09, 2.81	0.18 (0.52) − 0.85, 1.21
EG_int_ Intervention-only				
Block 1	3.79 (0.45) 2.89, 4.69	3.99 (0.62) 2.76, 5.23	− 1.37 (0.34) 0.70, 2.04	− .012 (0.32) − 0.75, 0.51
Block 2	2.63 (0.59) 1.45, 3.81	2.43 (0.72) 1.0, 3.86	2.22 (0.41) 1.38, 3.04	0.72 (0.50) − 0.27, 1.72
EG_combined_ Exclusion + Intervention				
Block 1	4.16 (0.46) 3.24, 5.08	4.85 (0.63) 3.59, 6.12	2.36, (0.34) 1.68, 3.05	0.43 (0.32) − 0.22, 1.07
Block 2	6.87 (0.60) 5.66, 8.07	7.67 (0.73) 6.21, 9.13	3.54 (0.42) 2.70, 4.38	3.05 (0.51) 2.04, 4.06

As for the late P3, the amplitude increase related to the reduced ball reception probability was mostly expressed in EG_combined_ (see Table 2). In EG_excl_, a slight increase was observed, whereas the P3 was reduced in block 2 in EG_int_. As for the early P3, no main effect of ‘block was obtained, F(1,72) = 3.29, *p* = 0.074, η_p_^2^ = 0.044, but a significant interaction with the group assignment, F(2,72) = 15.02, *p* < 0.001, η_p_^2^ = 0.294. Post-hoc comparisons focused on the differential expression of the P3 effect (block 2—block 1): The increase of the P3 from block 1 to block 2 in EG_combined_ was significantly larger as compared to EG_excl_, F(1,47) = 6.94, *p* = 0.011, η_p_^2^ = 0.129, and as compared to EG_int_, F(1,49) = 38.26, *p* < 0.001, η_p_^2^ = 0.438. Moreover, the effect differed between EG_excl_ and EG_int_, F(1,48) = 6.18, *p* = 0.016, η_p_^2^ = 0.114. As for the early P3, the critical interaction between ‘block’ and ‘group’ was not modulated by the position of the midline electrodes, F(2.96,106.6) = 1.36, *p* = 0.259, η_p_^2^ = 0.036.

A posteriori, the ERP effects were correlated with relevant scales of the self-reports: The expression of the early P3 effect was not correlated with the self-reported threat to belonging (r = −0.211, *p* = 0.069) or with the change in negative mood (r = 0.107, *p* = 0.360). The same held for the expression of the late P3 effect which was neither correlated with the self-reported threat to belonging (r = −0.204, *p* = 0.079) nor with the change in negative mood (r = 0.093, *p* = 0.478).

The a posteriori defined N2 component was not modulated by the experimental manipulation: Its amplitude was not significantly affected by the transition (factor ‘block’), F(1,72) = 3.25, *p* = 0.076, η_p_^2^ = 0.043, or by an interaction of transition and group assignment, F(2,72) = 0.96, *p* = 0.390, η_p_^2^ = 0.026.

### ERP effects: Ball reception of the intended co-player

To probe the expectancy of an intervention, ERP analysis focused on the event ‘ball reception of the intended co-player’, i.e., the trials in which the supervisor did not overrule the decision of the participant on the recipient of her/her ball throw. In block 2, this expected decisional autonomy was challenged in EG_int_ and EG_combined_.

Figure 2 (lower panel) depicts the grand-averaged ERP responses separated for the experimental groups. A transient negativity at 180 ms (N2) is immediately released by a transient positivity with a peak at 220 ms. Whereas a return to baseline level is observed in EG_excl_, a second positive deflection between 250 and 400 ms is expressed in EG_int_ and EG_combined_—predominantly in block 2. As mentioned above, the global field power of the difference waves (Δ(block 2—block 1)) indicated the first maximum at 220 ms followed by a local maximum at 370 ms. This second activation corresponds to the hypothesized P3 effect, and its expression was analyzed in the time range 340–400 ms. Moreover, the first negative (N2) and positive (P2) deflection were analyzed a posteriori.

The level of the P3 amplitude was affected differently by a transition-to-intervention depending on group assignment: Unlike the strong effect in EG_combined_, the increase was moderate in EG_int_. In EG_excl_—which retains control of decisions—the amplitude was not modulated. The ANOVA indicates a significant effect of ‘block’, F(1,72) = 11.66, *p* = 0.001, η_p_^2^ = 0.139, which was modulated by the factor ‘group’, F(2,72) = 9.85, *p* < 0.001, η_p_^2^ = 0.215. Post-hoc comparisons confirmed the increase of the P3 effect (block 2—block 1) when comparing EG_combined_ and EG_excl_, F(1,47) = 16.66, *p* < 0.001, η_p_^2^ = 0.262, and EG_combined_ and EG_int_, F(1,49) = 5.66, *p* = 0.021, η_p_^2^ = 0.103, respectively. A stronger expression of the P3-effect in EG_int_ as compared to EG_excl_ was also confirmed, F(1,48) = 5.55, *p* = 0.023, η_p_^2^ = 0.104. Please note, that the critical interaction of ‘block’ and ‘group’ was again not modulated by the position of the electrode, F(2.88,103.59) = 2.10, *p* = 0.107, η_p_^2^ = 0.055.

As for the P3 effect related to the event ‘ball reception of the participant’, the expression of the P3 effect related to the event ‘ball reception of intended co-player’ was not correlated with the self-reported threat to social control (r = 0.012, *p* = 0.920) or with the change in negative mood (r = 0.008, *p* = 0.946).

The a posteriori defined N2 component was affected by the transition to a social threat in block 2: Its amplitude was found to be significantly reduced (factor ‘block’), F(1,72) = 6.48, *p* = 0.013, η_p_^2^ = 0.083. However, this reduction was not modulated by the single or joint onset of the threats, F(2,72) = 0.89, *p* = 0.416, η_p_^2^ = 0.024. A similar pattern was observed for the a posteriori defined P2: The ANOVA revealed a significant effect of ‘block’, F(1,72) = 7.96, *p* = 0.006, η_p_^2^ = 0.100, here reflecting a significant increase in block 2. However, it was not significantly modulated by the factor ‘group’, F(2,72) = 2.85, *p* = 0.064, η_p_^2^ = 0.073.

In the “Supplementary material”, the results of an additional post-hoc analysis can be found: Based on previous results reporting an adaptation process of the P3 effect^11^, a split-half analysis was applied to test for a reduction of the P3 amplitude within block 2. The results provided evidence that the enhanced P3 amplitude in the group EG_combined_ is also associated with the absence of an adaptation process.

## Discussion

This research aimed to contribute to a better understanding of the interaction of different social threats. The self-report data showed a specific effect of exclusion and intervention on the corresponding scales ‘belonging’ and ‘control’, respectively. Crucially, the effects on both scales are enhanced by a joint onset of the threats. The predicted interaction is more clearly expressed in the ERPs: Here, the onset of a single specific threat triggers a P3-effect in corresponding probe events (exclusion: ‘ball reception: participant’, intervention: ‘ball reception: intended co-player’). Both P3 effects are significantly enhanced if participants were exposed to both threats. Subsequently, we will elaborate the implications of these findings.

### Manifestations of the interaction of different social threats on a neural level

The observed modulation of the P3 amplitude induced by the combination of threats confirms previous results, but—more importantly—sheds new light on the processing of social threats. First, the results support the notion that the P3-effect is not a mere reflection of probability, but rather reflects expression of the violations of subjective beliefs and expectations^11,26^. Second, the stronger expression of the P3-effect elicited by the joint onset of two threats can be attributed to a sensitization of a system detecting deviances in the expected participation and control. This sensitization contrasts the recently described adaptation linked to a ‘preexposure effect’^25^: Here, the previous experience of a social threat (loss-of-control) reduces the P3 response to a following social threat (exclusion). This reduction has been attributed to a faster adjustment of subjective expectations.

Sensitization in the detection of irregularities or inconsistencies has been assumed to serve the prediction of forthcoming events, such as surprise and belief updating^40^. According to a recently proposed framework on expectation updating and maintenance^41^, the updating process closes the gap to the experienced situational outcome and reflects an accommodation process. The latter can be linked to the adjustment of subjective expectancies^42,43^ and has also been observed in previous Cyberball studies^19^. Our results suggest that a joint onset of threats increases the sensitivity to expectancy violation. In contrast to the onset of a single threat, the adjustment process in a dual-threat situation appears to be delayed. This idea is supported by the additional split-half analysis indicating the absence of an adaptation process. A corresponding process can be triggered if the self-assignment of personal power is increased^11,26^. Although the specific neural implementation of these mechanisms remains to be identified with neuroimaging or neurostimulation techniques^44,45^, the current ERP effects are in line with the assumption of a common neural network processing inconsistencies, such as expectancy violations^16^.

Importantly, the effects can hardly be attributed to a superimposition of ERP components generated by different cognitive processes^46^. We can rule out that the onset of a specific threat will automatically increase the response to concurrent social events: In block 2, the P3-effect triggered by the event ‘ball reception of the participant' are reduced in EG_int_. The same applies for the event ‘ball reception of the intended co-player’ in EG_excl_. This reduction might be attributed to a re-allocation of attentional resources to the event associated with the selective threat. A similar effect has previously been described for the withdrawal from social cues^19^.

Notably, this aforementioned process, as well as the sensitization, do not affect earlier ERP components previously observed in cyberball studies^37^, such as the N2 and P2.

### Manifestations of the interaction of different social threats in self-reports

Although the interaction of threats is clearly expressed on a neural level, the effect is less obvious in the self-reports: Here, the effects of a single and a joint onset of threats cannot be differentiated for the corresponding scales of the need-threat-questionnaire (belonging, control). The difference between the methods can partially be attributed to the post-hoc evaluation of a preceding social interaction required in the self-reports. In contrast to the online monitoring via ERP, post-hoc self-reports are affected by several psychological mechanisms, such as social desirability^47^. Moreover, one has to consider that the relative judgment required (comparison of feelings in block 1 vs block 2) enhances an additional memory load and a more-controlled judgment. These factors account for the lack of a correlation between the expression of the ‘online’ P3-effect and the ‘offline’ self-reports.

Moreover, the current data also shed light on the relatedness of different scales of the questionnaire. The onset of exclusion induces a threat to ‘belonging’ which is also related to a threat to ‘control’. This spreading effect might signal that the experience of exclusion affects all fundamental needs^2^—or a lack of specificity in the self-reports scales^48^. Our correlation analysis suggests that the covariations are probably mediated by the increase in negative mood^49^. The onset of a single threat leads to an affective response which biases the response to other scales^25^. Notably, this mediation does not apply in the case of a joint onset of two threats. Here, the needs for ‘belonging’ and ‘control’ are selectively addressed by experimental manipulation, and self-reports on the scales are less affected by negative mood.

### Limitations

As with all empirical research, certain limitations of this study should be considered when interpreting the results. First, although the combination of two social threats is innovative and furthers our understanding of a common cognitive mechanisms, at present our conclusions are limited to the threats selected, namely exclusion and loss-of-control. As this restriction is due to the Cyberball setup, innovative experimental setups are called for that allow for an extension of the range of social threats studied. Second, participants were rather able to get prepared for the onset of an intervention than for the onset of exclusion because a ‘supervisor’ was already mentioned in the instruction. Although this information did not affect the expression of the P3 effect—and its adaptation (see Sect. “Supplementary material”)—it might contribute to the evaluation provided in the self-reports: According to the difference score (see Table 1), a threat to belonging is more expressed than to control in case of a joint onset. Third, the analysis was focused on the P3 effect, and did not explicitly consider ERP components previously related to the processing of exclusion^37^. Considering additional psychophysiological markers, one might be able to estimate the contribution of affective processes to the interaction process^50^. Finally, most of the participants can be related to a WEIRD social background^51^ which has to be considered. This necessity is further strengthened by the dominance of female participants in our sample. We cannot exclude that the sensitivity to the onset of joint threats is modulated by gender. To strengthen the generalizability of the results, a larger and more heterogenous sample would be required.

## Conclusions

In this first approach to studying the interaction of different social threats, we identified a sensitization process that facilitates the detection of deviances from an expected outcome. The results support the idea that social threats are defined as inconsistencies in a subjective belief system^16^, and can be integrated into recently proposed functional frameworks of expectancy update and maintenance^41^. Given the central role of social participation in mental health^52^, the interaction mechanism identified in the present research might contribute to the expression of clinical symptoms.

## Supplementary Information

Supplementary Information.

## Data Availability

All data (self-reports, pre-processed EEG data), program, and analysis codes are available in a data repository (https://osf.io/kad5t/?view_only=28a0f79c140f443aabaa2889bc3e296d).

## References

[1] Baumeister R, Leary M (1995). The need to belong: desire for interpersonal attachments as a fundamental human motivation. Psychol. Bull..

[2] Williams KD (2007). Ostracism. Annu. Rev. Psychol..

[3] Leotti LA, Iyengar SS, Ochsner KN (2010). Born to choose: The origins and value of the need for control. Trends Cogn. Sci..

[4] Proulx T, Inzlicht M (2012). The five “A” s of meaning maintenance: Finding meaning in the theories of sense-making. Psychol. Inq..

[5] Narayanan J, Tai K, Kinias Z (2013). Power motivates interpersonal connection following social exclusion. Organ. Behav. Hum. Decis. Process..

[6] Niedeggen M, Kerschreiter R, Hirte D, Weschke S (2017). Being low prepares for being neglected: Verticality affects expectancy of social participation. Psychon. Bull. Rev..

[7] Hudac CM (2019). Social priming modulates the neural response to ostracism: a new exploratory approach. Soc. Neurosci..

[8] Williams KD, Jarvis B (2006). Cyberball: A program for use in research on interpersonal ostracism and acceptance. Behav. Res. Methods.

[9] Hartgerink CH, Van Beest I, Wicherts JM, Williams KD (2015). The ordinal effects of ostracism: A meta-analysis of 120 Cyberball studies. PloS One.

[10] Inesi ME, Botti S, Dubois D, Rucker DD, Galinsky AD (2011). Power and choice: Their dynamic interplay in quenching the thirst for personal control. Psychol. Sci..

[11] Niedeggen M, Kerschreiter R, Schuck K (2019). Loss of control as a violation of expectations: Testing the predictions of a common inconsistency compensation approach in an inclusionary cyberball game. PLoS One.

[12] Williams KD (2009). Ostracism: A temporal need-threat model. Adv. Exp. Soc. Psychol..

[13] Park CL (2010). Making sense of the meaning literature: an integrative review of meaning making and its effects on adjustment to stressful life events. Psychol. Bull..

[14] Kay AC, Gaucher D, Napier JL, Callan MJ, Laurin K (2008). God and the government: testing a compensatory control mechanism for the support of external systems. J. Pers. Soc. Psychol..

[15] Heine SJ, Proulx T, Vohs KD (2006). The meaning maintenance model: On the coherence of social motivations. Pers. Soc. Psychol. Rev..

[16] Proulx T, Inzlicht M, Harmon JE (2012). Understanding all inconsistency compensation as a palliative response to violated expectations. Trends Cogn. Sci..

[17] Mwilambwe-Tshilobo L, Spreng RN (2021). Social exclusion reliably engages the default network: A meta-analysis of Cyberball. NeuroImage.

[18] Gutz L, Küpper C, Renneberg B, Niedeggen M (2011). Processing social participation: An event-related brain potential study. Cogn. Neurosci. Neuropsychol..

[19] Kawamoto T, Nittono H, Ura M (2013). Cognitive, affective, and motivational changes during ostracism: An ERP, EMG, and eeg study using a computerized cyberball task. Neurosci. J..

[20] Polich J (2007). Updating P300: an integrative theory of P3a and P3b. Clin. Neurophysiol..

[21] Weinbrecht A, Niedeggen M, Roepke S, Renneberg B (2018). Feeling excluded no matter what? Bias in the processing of social participation in borderline personality disorder. Neuroimage Clin..

[22] Weschke S, Niedeggen M (2015). ERP effects and perceived exclusion in the Cyberball paradigm: Correlates of expectancy violation?. Brain Res..

[23] Slepian ML, Masicampo E, Ambady N (2015). Cognition from on high and down low: Verticality and construal level. J. Pers. Soc. Psychol..

[24] Randles D, Inzlicht M, Proulx T, Tullett AM, Heine SJ (2015). Is dissonance reduction a special case of fluid compensation? Evidence that dissonant cognitions cause compensatory affirmation and abstraction. J. Pers. Soc. Psychol..

[25] Fang X, Yang Y-F, Kerschreiter R, Niedeggen M (2022). From loss of control to social exclusion: ERP effects of preexposure to a social threat in the cyberball paradigm. Brain Sci..

[26] Schuck K, Niedeggen M, Kerschreiter R (2018). Violated expectations in the cyberball paradigm: Testing the expectancy account of social participation with ERP. Front. Psychol..

[27] Gollier-Briant F (2016). Neural correlates of three types of negative life events during angry face processing in adolescents. Soc. Cogn. Affect. Neurosci..

[28] Erdfelder E, Faul F, Buchner A (1996). GPOWER: A general power analysis program. Behav. Res. Methods Instrum. Comput..

[29] Weschke S, Niedeggen M (2013). The effect of the physical presence of co-players on perceived ostracism and event-related brain potentials in the cyberball paradigm. PLoS One.

[30] Weschke S, Niedeggen M (2016). Target and non-target processing during oddball and cyberball: A comparative event-related potential study. PloS one.

[31] Marks DF (1973). Visual imagery differences in the recall of pictures. Br. J. Psychol..

[32] Peirce JW (2007). PsychoPy—Psychophysics software in Python. J. Neurosci. Meth..

[33] Lim S, Reeves B (2009). Being in the game: Effects of avatar choice and point of view on psychophysiological responses during play. Media Psychol..

[34] Williams KD, Cheung CK, Choi W (2000). Cyberostracism: Effects of being ignored over the Internet. J. Pers. Soc. Psychol..

[35] Watson D, Clark LA, Tellegen A (1988). Development and validation of brief measures of positive and negative affect: the PANAS scales. J. Pers. Soc. Psychol..

[36] Lammers J, Stoker JI, Stapel DA (2009). Differentiating social and personal power: Opposite effects on stereotyping, but parallel effects on behavioral approach tendencies. Psychol. Sci..

[37] Mills L (2023). A systematic review and meta-analysis of electrophysiological studies of online social exclusion: evidence for the neurobiological impacts of cyberbullying. Adolesc. Res. Rev..

[38] Sreekrishnan A (2014). Kin rejection: social signals, neural response and perceived distress during social exclusion. Dev. Sci..

[39] Weinbrecht A, Niedeggen M, Roepke S, Renneberg B (2021). Processing of increased frequency of social interaction in social anxiety disorder and borderline personality disorder. Sci. Rep..

[40] Visalli A, Capizzi M, Ambrosini E, Kopp B, Vallesi A (2021). Electroencephalographic correlates of temporal Bayesian belief updating and surprise. NeuroImage.

[41] Panitz C (2021). A revised framework for the investigation of expectation update versus maintenance in the context of expectation violations: the ViolEx 2.0 model. Front. Psychol..

[42] Mars RB (2008). Trial-by-trial fluctuations in the event-related electroencephalogram reflect dynamic changes in the degree of surprise. J. Neurosci..

[43] Kolossa A, Fingscheidt T, Wessel K, Kopp B (2013). A model-based approach to trial-by-trial P300 amplitude fluctuations. Front. Hum. Neurosci..

[44] Koslov K, Mendes WB, Pajtas PE, Pizzagalli DA (2011). Asymmetry in resting intracortical activity as a buffer to social threat. Psychol. Sci..

[45] Riva P, Romero Lauro LJ, DeWall CN, Bushman BJ (2012). Buffer the pain away: Stimulating the right ventrolateral prefrontal cortex reduces pain following social exclusion. Psychol. Sci..

[46] Rösler F, Heil M (1991). Toward a functional categorization of slow waves: taking into account past and future events. Psychophysiology.

[47] Latkin CA, Edwards C, Davey-Rothwell MA, Tobin KE (2017). The relationship between social desirability bias and self-reports of health, substance use, and social network factors among urban substance users in Baltimore, Maryland. Addict. Behav..

[48] Gerber J, Chang S-H, Reimel H (2017). Construct validity of Williams' ostracism needs threat scale. Pers. Indiv. Differ..

[49] Cohen LH, Towbes LC, Flocco R (1988). Effects of induced mood on self-reported life events and perceived and received social support. J. Pers. Soc. Psychol..

[50] Inagaki TK, Gianaros PJ (2022). Resting (tonic) blood pressure is associated with sensitivity to imagined and acute experiences of social pain: Evidence from three studies. Psychol. Sci..

[51] Henrich J, Heine SJ, Norenzayan A (2010). The weirdest people in the world?. Behav. Brain Sci..

[52] Reinhard MA (2020). The vicious circle of social exclusion and psychopathology: A systematic review of experimental ostracism research in psychiatric disorders. Eur. Arch. Psychiatry Clin. Neurosci..

