# Supplementary material for: Electrophysiological evidence for sensitization effects elicited by concurrent social threats
Source: Sci Rep. 2023 Jul 28;13:12285. doi: 10.1038/s41598-023-39456-0 (PMC10382520; doi:10.1038/s41598-023-39456-0)
Supplement: Supplementary file 1 — Supplementary Information. [file 41598_2023_39456_MOESM1_ESM.docx]

**Supplement 1: Post-hoc analysis of the split-half effects in block 2**

The additional analysis was done post-hoc to consider the dynamics of the P3 response in block 2. Its rationale was the identification of adaptation effects by means of a split-half analysis.

Methods

ERP segments were separately averaged within the first and second half of the second experiment block. Averaged data were based on the artifact-free set of preprocessed single trials and included at least ten single trials. In case that the data set of an individual comprised less than 20 single trials, a partial overlap was allowed (e.g.: for 17 trials, the first half was based on trial 1-10, and the second in trial 8-17). The partial overlap is a more-conservative approach with respect to the supposed effect, and strengthen the reliability of the ERPs.

For each half, the mean amplitudes in the time range of the late P3 component were computed (ball reception/participant: 360-420 ms, ball reception/intended co-player: 340-400 ms). Two statistical analysis were performed: The first one focused on the effect ‘ball reception/participant’, and comprised the within-participant factors *half* (first vs. second) and the between-participant factor *group assignment* (EG_excl_ vs EG_combined_). The second analysis focused on the effect ‘ball reception/intended co-player’, and comprised the within-participant factors *half* (first vs. second) and the between-participant factor *group assignment* (EG_int_ vs EG_combined_).

Results

**Adaptation in the processing of a transition-to-exclusion in EG_excl_ and EG_combined_:** The P3 amplitude to the event ‘ball reception of the participant’ showed a diametrical pattern (see Table). In the case of the onset of the single threat (EG_excl_), the P3 amplitude was reduced in the second half, whereas the joint onset of two threats (EG_combined_) lead to an increase in the P3 amplitude. The significant interaction of the factors ‘group’ and ‘block half”, F(1,47)=4.25, p=.045, η_p_²=.083, was not modulated by electrode position F(1.33, 62.30)=2.11, p=0.145, η_p_²=.043.

**Adaptation in the processing of a transition-to-intervention in EG_int_ and EG_combined_:** The corresponding probe - the P3 amplitude to the event ‘ball reception of the co-player’ – showed a reduction over time within block 2, F(1,48)=13.36, p<.001, η_p_²=.043. This effect of the factor ‘half’ was not significantly modulated by group assignment, F(1,48)=0.01, p=.935, η_p_²=.000.

The data indicate that the adaptation cannot be observed for the processing of exclusionary signals – if they are accompanied by a second social threat.

Table 1: Split-half effects of the P3 amplitude in block 2. The values refer to the mean amplitude (in mV), standard deviation (in brackets) and the confidence limits. Amplitude reductions were consistently expressed – with exception to the processing of the transition-to-exclusion in the group EG_combined_

|  |  | ***P3-amplitude in block 2*** | |
| --- | --- | --- | --- |
|  |  | *First Half* | *Second Half* |
| *Transition-to-exclusion*  *Probe: “Ball reception participant’*  *Mean amplitude 360-420* | *EG_EXCL_* | *4.44 (.93)*  *2.57, 6.31* | *3.44 (.90)*  *1.65, 5.23* |
|  | *EG_COMBINED_* | *6.00 (.91)*  *4.15, 7.82* | *7.55 (.87)*  *5.80, 9.30* |
| *Transition-to-intervention*  *Probe: “Ball reception co-player’*  *Mean amplitude 340-400* | *EG_INT_* | *1.42 (.66)*  *.09, 2.76* | *-.09 (.64)*  *-1.38, 1.20* |
|  | *EG_COMBINED_* | *3.73 (.69)*  *2.34, 5.11* | *2.28 (.67)*  *.94, 3.62* |

**Supplement 2: Reliability of the ERP effects**

The averaged ERPs in block 1 and block 2 rely on different numbers of trials: Due to the experimental design (transition-to-exclusion and transition-to-intervention), less trials are available for averaging in the second block. To account for these differences, the signal-to-noise ratio of ERPs between the blocks was matched. To this end, the number of EEG segments in the condition “ball reception” in block 1 was adjusted to the number of segments in the condition “ball reception” in block 2 by random selection. This procedure was also applied to the condition ‘ball reception of the intended co-player’. This allows us to test whether the statistical effects can be confirmed if the signal-to-noise ratio is reduced – by increasing the variance in block 1.

The grand-averaged data (see figure below) revealed that the overall pattern remains comparable. More importantly, this also applies to critical statistical interactions:


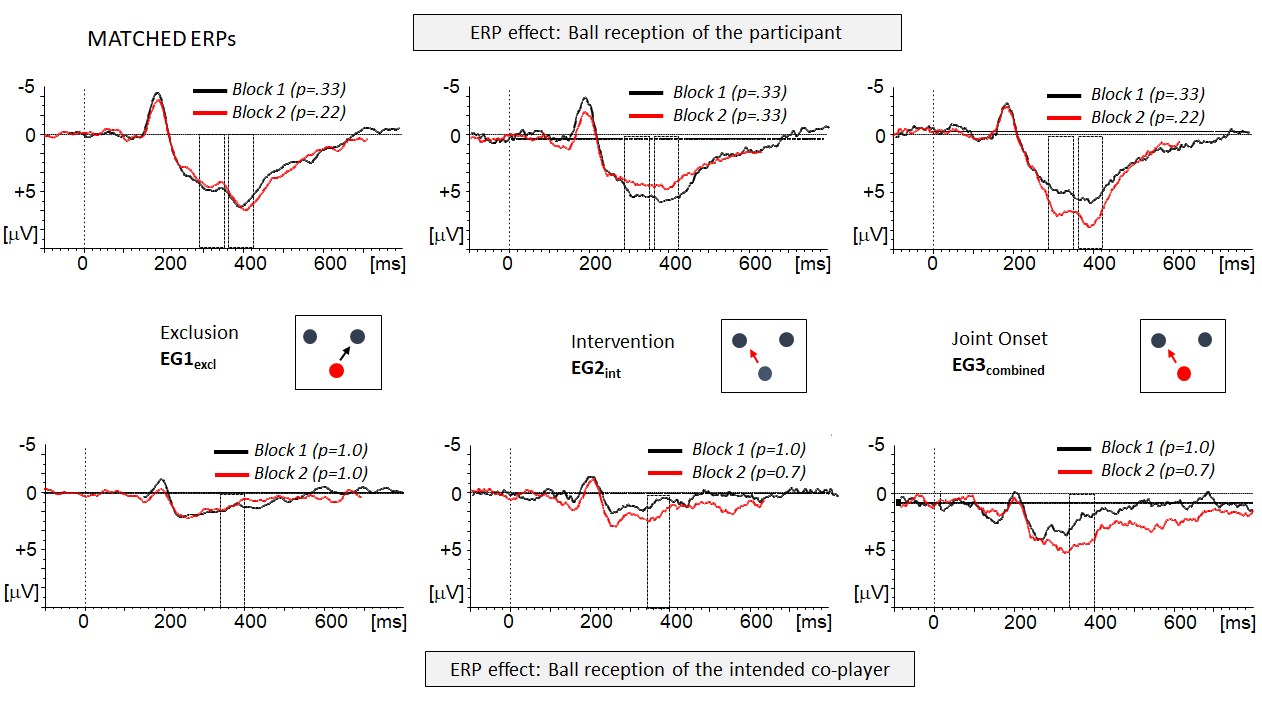


Figure 1: Grand-averaged potentials according to Figure 2 (see manuscript). The number of trials between conditions has been matched.

Table 2: Comparison of statistical effects based on non-matched (see manuscript) and matched trials.

|  | ERPs: Ball reception of participant | | ERP: Ball reception intended co-player |
| --- | --- | --- | --- |
|  | 290-350 ms | 360-420 ms | 340-400 ms |
| Statistics:  Non-matched for the number of trials  (see manuscript) | Group x Block  F(2,72)=18.68, p<0.001, **η_p_²**=.342 | Group x Block  F(2,72)=15.02, p<0.001, **η_p_²**=.294 | Group x Block  F(2,72)=9.85, p<0.001, **η_p_²**=.215 |
| Statistics:  Matched for the number of trials | Group x Block  F(2,72)=15.96, p<0.001, **η_p_²**=.307 | Group x Block  F(2,72)=11.68, p<0.001, **η_p_²**=.254 | Group x Block  F(2,72)=6.00, p=0.004, **η_p_²**=.143 |

Please note, that the subsequent pairwise comparisons also indicated that the differences between the groups were due to a selective enhancement in P3 amplitude in the EEG_combined_ group.
